# Capacity adiposity indices to identify metabolic syndrome in subjects with intermediate cardiovascular risk (MARK study)

**DOI:** 10.1371/journal.pone.0209992

**Published:** 2019-01-25

**Authors:** Manuel A. Gomez-Marcos, Leticia Gomez-Sanchez, Maria C. Patino-Alonso, Jose I. Recio-Rodriguez, Marta Gomez-Sanchez, Fernando Rigo, Ruth Marti, Cristina Agudo-Conde, Jose A. Maderuelo-Fernandez, Rafel Ramos, Luis Garcia-Ortiz, Emiliano Rodriguez-Sanchez

**Affiliations:** 1 Institute of Biomedical Research of Salamanca (IBSAL), Primary Health Care Research Unit, the Alamedilla Health Center, Salamanca, Spain; 2 Health Service of Castilla y León (SACyL), Salamanca, Spain; 3 Department of Medicine, University of Salamanca, Salamanca, Spain; 4 Department of Statistics, University of Salamanca, Salamanca, Spain; 5 Department of Nursing and Physical Therapy, University of Salamanca, Salamanca, Spain; 6 San Agustín Health Care Center, Department of Health, Balearic Islands (IBSALUD), Palma de Majorca, Spain; 7 Girona Research Unit, Primary Care Research Institute Jordi Gol (IDIAP Jordi Gol), Biomedical Research Institute of Girona Dr. Josep Trueta (IDBGI), Girona, Spain; 8 Department of Medical Sciences, University of Girona, Girona, Spain; 9 Department of Biomedical Sciences and Diagnosis, University of Salamanca, Salamanca, Spain; Beijing Key Laboratory of Diabetes Prevention and Research, CHINA

## Abstract

**Background:**

Obesity increases mortality, and is linked to cardiovascular diseases and metabolic syndrome (MetS). Therefore, the purpose of this study was to analyze the ability of different adiposity indices to identify subjects with MetS among people with intermediate cariovascular risk.

**Materials and methods:**

The cross-sectional study involved 2478 subjects, recruited by the MARK study. Adiposity measures: general adiposity by body mass index (BMI), central adiposity by waist-to-height ratio (WHtR), fat mass percent by the Clínica Universidad de Navarra—body adiposity estimator (CUN-BAE), percentage of body fat and of visceral adipose tissue by body roundness index (BRI) and visceral obesity and general adiposity with body shape index (ABSI). The diagnosis of MetS was made in accordance with the criteria established in the international consensus of the Joint Scientific Statement National Cholesterol Education Program III.

**Results:**

The highest correlation coefficients were obtained by the glycemic components (HbA1c and FPG) of the MetS and ranged from 0.155 to 0.320. The exception was ABSI, which showed lower values in the global analysis and in the males. Values of the area under the ROC curve with the adiposity indices ranged from 0.773 with the BMI in males to 0.567 with ABSI in males. In the logistic regression analysis, all adiposity factors, except ABSI, showed similar OR values of MetS after adjusting for possible confounding factors. In the global analysis, the adiposity index that showed a highest OR of MetS was CUN-BAE (OR 5.50; 95% CI 4.27–7.09). In the analysis by gender, the highest ORs were BMI in males (OR 5.98; 95% CI 4.70–7.60) and both WHtR and BRI in females (OR 4.15; 95% CI 3.09–5.58).

**Conclusion:**

All adiposity indices, except for ABSI, show an association with MetS and similar ability to detect subjects with MetS among people with intermediate cariovascular risk.

## Introduction

Obesity increases mortality [1], and is linked to cardiovascular diseases and metabolic syndrome (MetS) [2, 3]. Similarly, MetS multiplies the risk of cardiovascular disease by 2 and the risk of mortality from all causes by 1.5 [2]. It is also known that the complications associated with obesity are due, above all, to excess adiposity rather than weight gain [4]. In this context, the most objective way to determine adiposity is by direct measurement of the body fat percentage through computed tomography or magnetic resonance imaging [5]. However, the high cost and limited accessibility of these tests hinder their use in daily clinical practice. To alleviate these limitations, an indirect measurement of adiposity was designed in Spain, the CUN-BAE (Clínica Universidad de Navarra—Body Adiposity Estimator). This measure estimates the percentage of body fat, incorporating information on sex and age, and has shown a greater association with cardiovascular risk factors than the body mass index (BMI) [6].

Furthermore, classic adiposity indices such as the BMI or waist circumference (WC) [7] have important limitations. The BMI, for example, does not distinguish between lean mass and fat mass, nor does it discriminate the location of central/peripheral fat [8], while WC does not take into account the height and weight of the subject [9] and can over- or underestimate obesity in tall or short individuals [10]. An alternative which can mitigate this limitation is to use the waist-to-height ratio (WHtR) as an indicator of abdominal adiposity [11] since the association with cardiovascular risk factors is greater than with WC [12–14].

Due to the limitations of the classic measures used to assess obesity, new adiposity indices have been developed that take WC into account in addition to weight and/or height. Thus, in 2012 Krakauer et al [15] proposed the body shape index (ABSI), which allows visceral obesity and general adiposity to be estimated from easy-to-obtain clinical parameters (height, weight and WC), and which has been shown to predict mortality independently of BMI [15–17]. Likewise, in 2013 Thomas et al [18] developed the body roundness index (BRI), which combines height and WC and was able to estimate the percentage of body fat and visceral adipose tissue. The BRI has shown greater predictive capacity for diabetes and hypertension than other measures of adiposity [19, 20].

The relationship between CUN-BAE and classic adiposity indices such as BMI and WHtR, and new indices such as ABSI and BRI has not been studied. Moreover, although there are publications on the association between indices of adiposity and MetS, there is no consensus on which is the best index to identify subjects with MetS [8, 12 – 15].

The objective of this study was to analyze the ability of different adiposity indices to identify subjects with MetS among people with intermediate cariovascular risk.

## Materials and methods

### Study design

The results presented in this study correspond to a sub analysis of the data collected in the baseline visit of the *improving interMediAte RisK management (MARK)* study (NCT01428934) [21]. Trial Registration Clinical Trials.gov Identifier: https://clinicaltrials.gov/ct2/show/NCT01428934. Registered 2 September 2011. Last updated September 8, 2017. The characteristics of the subjects and their selection process, as well as the methodology of the measurements made with the subjects included in the study have been described in detail in the study protocol [21] and in the manuscripts presented by the MARK study group [11, 22].

### Ethics statement

Before inclusion in the study, all participants were informed about its objectives, the tests that were to be performed and the need to sign consent in order to participate. The study was approved by the research ethics committees of the Jordi Gol Institute for Primary Care Research (IDIAP Jordi Gol) and the health areas of Salamanca and Palma de Mallorca. The study was carried out in accordance with the recommendations of the Declaration of Helsinki.

### Characteristics and participants

The selection of subjects in this multicentre study was carried out by random sampling of individuals attending family medicine consultations in six primary care centers of three Spanish Autonomous Communities between July 2011 and June 2013 and meeting the inclusion criteria. The 2495 subjects recruited were aged 35 to 74 and had an intermediate cardiovascular risk as defined by one of the following criteria: 10-year coronary risk of 5–15% according to the REGICOR scale [23]; 10-year vascular mortality risk of 1–5% according to SCORE [24]; or moderate risk according to the guidelines of the European Society of Hypertension and Cardiology [25]. Exclusion criteria were: subjects with a history of atherosclerotic disease, terminal illness or being institutionalized at the time of the visit. This study examined 2478 of the 2495 subjects registered for the MARK study. We excluded 17 subjects who did not have any of the anthropometric measurements necessary to calculate the adiposity indices used in this study.

### Variables and measurement instruments

A detailed description of the procedures for the collection of clinical data, anthropometric measurements and laboratory tests performed has been previously published [21].

### Adiposity indices

#### Body weight and height

Two weight measurements were taken with an approved and calibrated electronic scale (accuracy ± 0.1 kg) (Seca 770, Medical scale and measurement systems, Birmingham, United Kingdom). Height was measured twice with a stadiometer (Seca 222). As reference measures of weight and height the mean of the two respective measurements was used. The BMI was calculated by dividing the weight in kg by height in m^2^. A value of BMI ≥ 30 kg/m^2^ was taken to define obesity.

#### Waist circumference and waist to height ratio

WC was measured following the 2007 recommendations of the Spanish Society for the Study of Obesity [26]. A value of WC ≥ 88 cm in females and ≥ 102 cm in males was taken to define abdominal obesity.

The WHtR was calculated by dividing WC in cm by height in cm [7, 27]. A value of WHtR: ≥ 0.604 cm/cm in females and ≥ 0.617 cm/cm in males was taken to define obesity.

#### University of Navarra Clinic—Body Adiposity Estimator (CUN-BAE)

The percentage of body fat was estimated following the recommendations of Gomez-Ambrosi et al [6] with the following formula, body fat percentage = -44.988 + (0.503 × age) + (10.689 × gender) + (3.172 × BMI)—(0.026 × BMI^2^) + (0.181 × BMI × gender)—(0.02 BMI × age)—(0.005 × BMI^2^ × gender) + (0.00021 × BMI^2^ × age) where age was in years, and gender was coded as 0 for males and 1 for females. A value of CUN-BAE ≥ 35 in females and ≥ 30 in males was taken to define increase in the percentage of body fat.

#### Body roundness index (BRI)

The BRI was calculated using the formula

$$BRI=364.2-365.5X\sqrt{1-((WC/{\left(2\pi \right)}^{2}/{\left(0.5height\right)}^{2})}$$

[28].

#### A body shape index (ABSI)

A Body Shape Index (ABSI) was based on WC adjusted for height and weight:

$$ABSI=WC/BMI^{2/3}Xheight{)}^{1/2}$$

[29].

### Diagnostic criteria of MetS

Following the criteria established by the international consensus of the Joint Scientific Statement National Cholesterol Education Program III [30], MetS was defined as the presence of three or more of the following five components: abdominal obesity, WC ≥ 88 cm in females and ≥ 102 cm in males; triglycerides ≥ 150 mg/dL or drug treatment for elevated triglycerides; high-density lipoprotein (HDL) cholesterol < 40 mg/dL in males or < 50 mg/dL in females; high blood pressure, systolic blood pressure (SBP) ≥ 130 mmHg or diastolic blood pressure (DBP) ≥ 85 mmHg or antihypertensive drug treatment and fasting plasma glucose (FPG) ≥ 100 mg/dL or drug treatment for elevated glucose.

### Laboratory determinations

Venous blood samples were taken between 08:00 and 09:00, after subjects had fasted for the previous 12 hours. Fasting plasma glucose, HbA1c, total serum cholesterol, HDL cholesterol and triglyceride levels were measured using automated standard enzymatic methods. Low-density lipoprotein (LDL) cholesterol was estimated using the Friedewald equation, LDL-Cholesterol = ((TC-(HDL-C+TG/5) for subjects with triglycerides levels < 400 mg/Dl.

### Office or clinical blood pressure

Three SBP and DBP measurements were performed with a OMRON model M10-IT validated sphygmomanometer (Omron Health Care, Kyoto, Japan), with the mean of the last two measures taken being registered. The measurement was made in accordance with the recommendations of the European Society of Hypertension.

### Smoking status

Smoking status (smoker/non-smoker) was recorded, with those who currently smoke or who quit smoking during the previous year considered smokers.

### Alcohol consumption

The amount of alcohol drunk in a week was recorded with a structured questionnaire and was measured in grams/week.

### Physical activity

We use the Minnesota questionnaire to measure physical activity in free time[31]. The questionnaire has been previously validated in both sexes in Spain. The questionnaire was collected by interviewers previously trained. They collected the physical activity carried out during the previous year, recording the type of activity and the duration of it. Each physical activity has a code of intensity, which is based on the quotient between the metabolic rate during the practice of physical activity and the basal metabolic rate (MET). 1 MET is equivalent to approximately 1 kcal / min of energy expenditure. Thus, we calculate the total energy expenditure in free time of total physical activity (EEPA_t) in kilocalories per week. Using the physical activity intensity code, we were also able to quantify the energy expenditure in physical activity (EEPA) according to the activity classification as intense, moderate, or light intensity as follows: light intensity was below 4 METs, such as walking (EEPA _light_). Moderate PA intensity was 4–5.5 METs, such as brisk walking (EEPA _moderate_). Intense PA intensity was greater than or equal to 6 METs, such as jogging (EEPA _intense_). Thus, for each particular subject: EEPA _total_ = EEPA _light_ + EEPA _moderate_ + EEPA _intense_ [32].

Additionally, in accordance with the recommendations of the American Heart Association [33], we considered participants to be sedentary if they do not meet the recommendations of moderate-intensity aerobic PA practice for a minimum of 30 min on 5 days each week (EEPA _moderate_ < 675 kcal/week) or high-intensity aerobic PA practice for a minimum of 20 min on 3 days each week (EEPA _intense_ < 420 kcal/week) [32].

### Recording diet

The adherence to the Mediterranean diet questionnaire used in the PREDIMED study was administered [34]. This questionnaire with 14 items related to compliance with different aspects of the Mediterranean diet has been validated in Spain. A score ≥ 9 is required to be considered good compliance.

All assessments were made within a period of 10 days.

### Statistical analysis

Continuous variables were presented as means ± standard deviation for normally distributed variables or medians (interquartile ranges) for the skewed variables. Statistical normality was tested using the Kolmogorov-Smirnov test. The means of two groups were compared using Student’s t-test or Mann-Wittney U test. All categorical variables were presented as numbers and proportions. Chi-square and Fisher’s exact tests were used for analysis of proportion.

The Spearman rho correlation coefficient was calculated to measure the CUN-BAE relationships with other measures of adiposity, as well as the correlation between measures of adiposity with the components that make up the MetS.

Receiver-operating characteristic (ROC) analyses were performed to examine the ability of the different measures of adiposity to diagnose MetS. The area under the ROC curve (AUROC) and the 95% confidence intervals (CIs) were computed to compare the discriminative power of each adiposity index.

The Kappa coefficient was used to analyze the agreement between the different adiposity indices, taking the optimal cut points calculated in the ROC curves to identify the presence or absence of MetS.

To estimate the association of the presence of adiposity, defined by the different adiposity indices, with the presence de MetS, three logistic regression models were applied: model 1 adjusted for age (years) and gender (0 = male and 1 = female); model 2 further adjusted for smoking status (0 = non-smoker and 1 = smoker), physical activity status (0 = sedentary and 1 = active), adherence to the Mediterranean diet (0 = non-adherence and 1 = adherence) and alcohol consumption en gr/week; and model 3 further adjusted for antihypertensive drugs (0 = no and 1 = yes), lipid lowering drugs (0 = no and 1 = yes) and antidiabetic drugs (0 = no and 1 = yes).

Analyses were performed with the subjects overall and by gender. Data were analyzed using SPSS Statistics for Windows version 23.0 (IBM Corp, Armonk, NY). Values of p < 0.05 were considered statistically significant.

## Results

### Characteristics of the study subjects

Table 1 shows the general characteristics of the population studied, globally and by gender. Males made up 61.3% of the sample, the median age was 62 (56–67) years. All the adiposity indexes showed higher values in the females except the ABSI that was higher in the males. The mean values of SBP, DBP and triglycerides were higher in males, and of total-cholesterol, LDL-cholesterol, HDL- and HA1c higher in females. The prevalence of MetS in the study participants was 45.2% (42.6% in men and 49.4% in women; p = 0.001). The percentage of subjects with hypertension was higher in men, and the percentages of subjects with diabetes mellitus, obesity and sedentary behavior were higher in females.

**Table 1:** Baseline characteristics and of study subjects.

Variables	Global n = 2478	Males n = 1520 (61.3%)	Females n = 958(38.7%)	p Value
**Lifestyle factors (median IR o number and %)**				
Age (years)	62 (56–67)	62 (55–68)	62 (57–67)	0.252
Smoking	248 (37.7)	195 (29.8)	214 (32.5)	<0.001
Total physical activity (MET)	1800 (750–3293)	2064 (917–3836)	600 (750–2514)	0.011
**Anthropometric measures (median IR)**				
Height (m)	1.65 (1.57–1.71)	1.70 (1.65–1.74)	1.56 (1.52–1.60)	<0.001
Weight (kg)	78.2 (69.9–88.0)	82.0 (74.8–91.0)	70.8 (62.2–81.0)	<0.001
WC (cm)	100 (97–107)	102 (96–108)	100 (97–107)	<0.001
**Adiposity index (median IR)**				
CUN-BAE (%)	35.8 (34.4–41.4)	30.8(28.3–33.8)	42.8(39.4–46.9)	<0.001
BMI (kg/m^2^)	28.7 (26.3–31.7)	28.3 (26.3–33.8)	28.7 (26.3–31.7)	<0.001
WHtR (cm/cm)	0.61 (0.57–0.66)	0.60 (0.57–0.64)	0.62 (0.57–0.67)	<0.001
BRI	5.6 (4.7–6.7)	5.5 (4.7–6.4)	5.9 (4.7–7.2)	<0.001
ABSI	0.083 (0.080–0.086)	0.084 (0.082–0.086)	0.082 (0.079–0.086)	<0.001
**Cardiovascular risk factors (median IR)**				
SBP (mmHg)	136 (125–147)	137 (127–146)	133 (122–145)	<0.001
DBP (mmHg)	84 (78–91)	85 (79–92)	82 (76–89)	<0.001
Total-Cholesterol (mg/dl)	224 (198–250)	220 (193–245)	232 (205–260)	<0.001
LDL- Cholesterol (mg/dl)	140 (116–163)	138 (115–161)	142 (117–167)	0.010
HDL-Cholesterol (mg/dl)	48 (41–56)	46 (40–54)	50 (44–58)	<0.001
Triglycerides (mg/dl)	124 (124–174)	126 (191–179)	121 (92–166)	0.026
FPG (mg/dl)	98 (88–115)	98 (88–114)	97 (87–117)	0.451
HA1c (%)	5.8 (5.4–6.3)	5.7 (5.4–6.3)	5.8 (5.5–6.5)	<0.001
**Prevalence of cardiovascular risk factors, number and %)**				
Hypertension	1798 (72.6)	1169 (76.9)	629 (65.7)	<0.001
Diabetes Mellitus	843 (34.0)	491 (32.3)	352 (37.7)	0.013
MetS	1120 (45.2)	647 (42.6)	473 (49.4)	0.001
High BMI	907 (36.6)	512 (33.7)	395 (41.2)	<0.001
High WC	1559 (62.9)	800 (52.6)	759 (79.2)	<0.001
High WHtR	2396 (96.7)	1478 (97.2)	918 (95.8)	0.037
High CUN-BAE	2312 (93.3)	1404 (92.4)	908 (94.8)	0.011
Sedentary	1097 (44.3)	576 (37.9)	521 (54.4)	<0.001
**Drug treatment, n (%)**				
Antihypertensive drugs	1275 (51.5)	765 (50.3)	510 (53.2)	0.086
Antidiabetic drugs	514 (20.7)	288 (18.9)	226 (23.6)	0.003
Lipid-lowering drugs	672 (27.1)	385 (25.3)	287 (30.0)	0.007

Continuous variables were presented as medians (interquartile ranges) for the skewed variables. Categorical variables were presented as numbers and proportions.

We consider High BMI ≥ 30 kg/m^2^; High WC: WC ≥ 88 cm in females and ≥ 102 cm in males; High WHtR: ≥ 0.604 cm/cm in females and ≥ 0.617 cm/cm in males; High CUN-BAE ≥ 35 in females and ≥ 30 in males.

Abbreviations: IR, interquartile range. MET, basal metabolic rate. BMI, body mass index. WC, Waist circumference. CUN-BAE, Clínica Universidad de Navarra—Body Adiposity Estimator. WHtR, waist-to-height ratio. BRI, body roundness index. ABSI, a body shape index. SBP, systolic blood pressure. DBP, diastolic blood pressure. LDL, low density lipoprotein. HDL, high density lipoprotein. FPG, fasting plasma glucose. HbA1C, glycosylated hemoglobin. MetS, Metabolic syndrome. CVD RF, cardiovascular risk factors

p value differences between males and females.

### Correlations between adiposity indices and MetS components

Table 2 shows the Spearman correlation coefficients between adiposity indices and MetS components, global and stratified by gender. The highest correlation coefficients were obtained by the glycemic components (Hb A1c and FPG) of the MetS and ranged from 0.155 to 0.320. The exception was ABSI, which showed lower values in the global analysis and in the males.

**Table 2:** Spearman correlation coefficient between adiposity index and components of metabolic syndrome.

	CUN-BAE	BMI	WHtR	BRI	ABSI
**Global n = 2478**					
SBP	-0.028	0.101**	0.101**	0.082**	0.059*
DBP	-0.025	0.160**	0.070**	0.062**	0.049*
FPG	0.155**	0.260**	0.279**	0.214**	0.062**
HbA1c	0.212**	0.241**	0.287**	0.243**	0.064**
HDL-Cholesterol	0.030	-0.207**	-0.135**	-0.110**	-0.005
Triglycerides	0.078**	0.215**	0.164**	0.107**	-0.026
**Males n = 1520**					
SBP	0.128**	0.115**	0.115**	0.100**	0.027
DBP	0.124**	0.178**	0.088**	0.093**	-0.062*
FPG	0.197**	0.196**	0.264**	0.204**	0.047
HbA1c	0.256**	0.242**	0.255**	0.221**	0.061*
HDL-Cholesterol	-0.196**	-0.222**	-0.139**	-0.141**	-0.105**
Triglycerides	0.139**	0.191**	0.115**	0.086**	-0.097**
**Females n = 958**					
SBP	0.118**	0.099**	0.121**	0.117**	0.111**
DBP	0.133**	0.151**	0.079*	0.120*	0.074*
FPG	0.273**	0.253**	0.302**	0.218**	0.231**
HbA1c	0.277**	0.265**	0.320**	0.236**	0.254**
HDL-Cholesterol	-0.189**	-0.211**	-0.191**	-0.162**	-0.143**
Triglycerides	0.246**	0.255**	0.249**	0.180**	0.180**

Adiposity index with highest correlation coefficient for each variable in **bold**.

Abbreviations: CUN-BAE, Clínica Universidad de Navarra—Body Adiposity Estimator. BMI, body mass index. WHtR, waist-to-height ratio. BRI, body roundness index. ABSI, a body shape index. SBP, systolic blood pressure. DBP, diastolic blood pressure. FPG, fasting plasma glucose. HbA1C, glycosylated hemoglobin. HDL, high density lipoprotein.

Spearman Correlation

* p<0.05,

**p<0.01.

### Correlations of CUN-BAE with other indices of global adiposity and by gender

**Fig 1:**
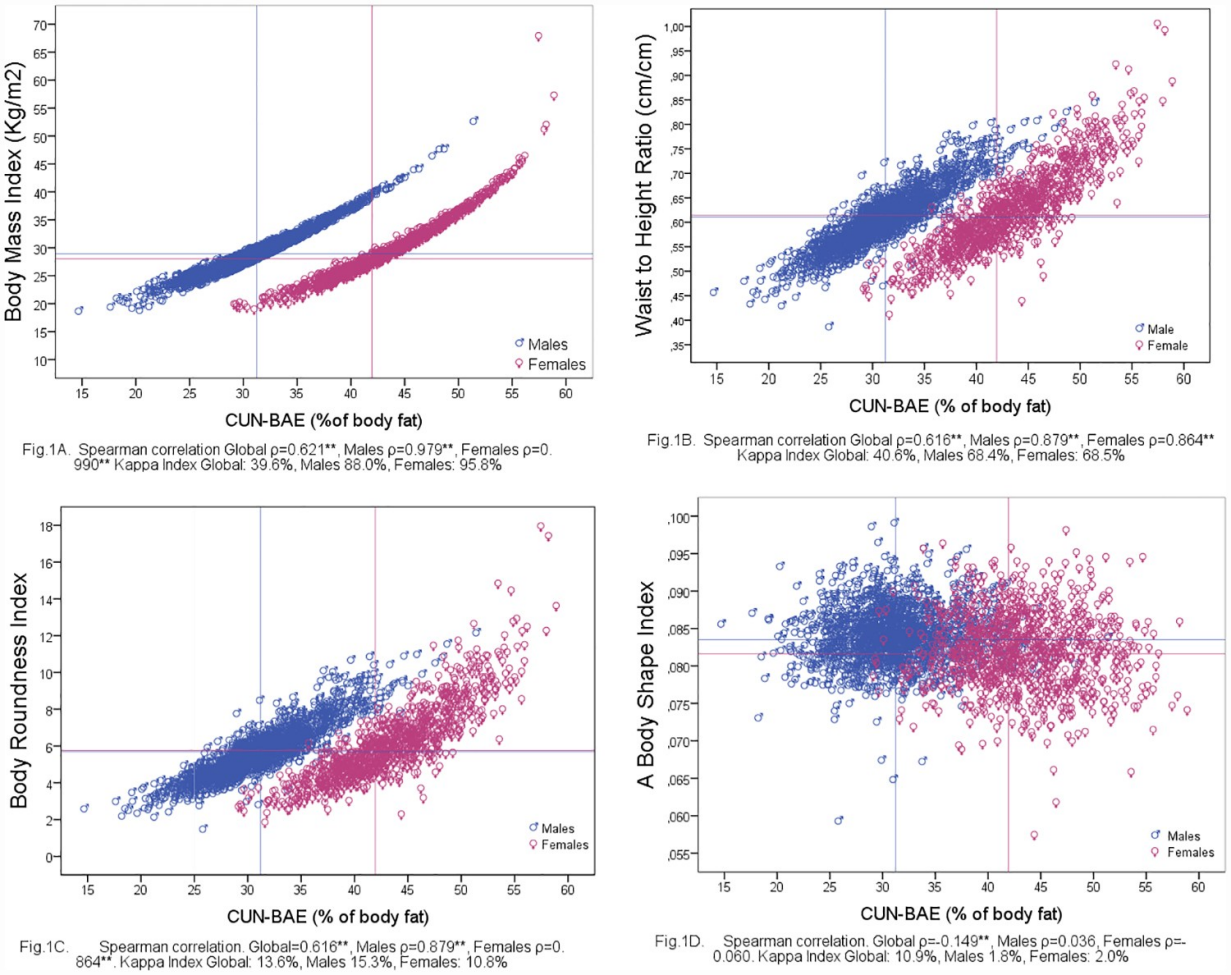
Correlation between CUN-BAE and BMI (Fig 1A), WHtR (Fig 1B), BRI (Fig 1C) and ABSI (Fig 1D) stratified by gender. Vertical lines indicate cut-offs for defining MetS according to CUN-BAE (31.22 males and 41.95 females respectively) and horizontal lines indicate cut-offs for defining MetS according to BMI (28.96 males and 28.02 females respectively), WHtR (0.611 males and 0.615 females respectively), BRI (5.69 males and 5.77 females respectively) and ABSI (0.0835 males and 0.0816 females respectively). In the lower part of the figure coefficient global Spearman correlation and by gender. ** p <0.001 Abbreviations: CUN-BAE, Clínica Universidad de Navarra—Body Adiposity Estimator. WHtR, waist-to-height ratio. BRI, body roundness index. ABSI, a body shape index.

The following Spearman correlation coefficients between the CUN-BAE and the different adiposity measurements were recorded: ρ = 0.621 with BMI; ρ = 0.616 with WHtR and BRI and ρ = -0.149 with ABSI. All coefficients increased when calculated by gender, except in the case of CUN-BAE with ABSI. Fig 1 also shows the dispersion diagrams by gender between CUN-BAE and BMI (Fig 1A), WHtR (Fig 1B), BRI (Fig 1C) and ABSI (Fig 1D).

### Capacity of the adiposity indices to predict MetS using AUROC analysis

**Fig 2:**
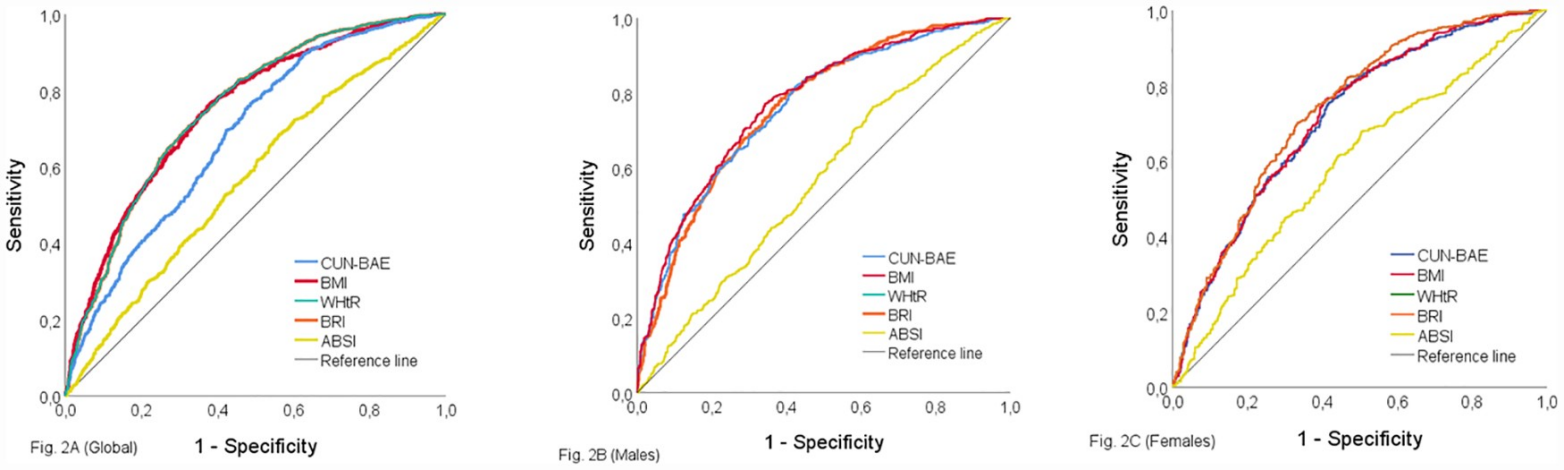
Receiver operating characteristic curve of CUN-BAE, BMI, WC, BRI and ABSI to identify subjects with metabolic syndrome, (A) global, (B) males and (C) females. Areas under the ROC curves are summarized in Table 3. Abbreviations: CUN-BAE, Clínica Universidad de Navarra—Body Adiposity Estimator. BMI, body mass index. WC, Waist circumference. BRI, body roundness index. ABSI, a body shape index.

Table 3 and Fig 2, Fig 2A (global), Fig 2B (males) and Fig 2C (females) show the AUROC values and the optimal cut-off points between MetS and the anthropometric measures, both globally and by gender. AUROC values were similar in all adiposity indices studied except in the case of ABSI, where they were lower. The highest values of the Youden index were obtained with the BMI (0.416) among males; and with the WHtR and the BRI in both the global analysis (0.380 in both cases), and among females (0.368 in both cases).

**Table 3:** Adjusted area under receiver operating characteristic curve and cut-off points, sensitivity and specificity for the various adiposity index and metabolic syndrome.

	ROC (95% CI)	Cut-off	Sensitivity (%)	Specificity (%)	Youden index
**Global n = 2478**					
CUN-BAE	0.684 (0.663–0.704)	33.15	0.700	0.592	0.292
BMI	0.748 (0.729–0.767)	28.73	0.692	0.680	0.372
WHtR	**0.752 (0.733–0.771)**	0.61	0.695	0.685	**0.380**
BRI	**0.752 (0.733–0.771)**	5.67	0.695	0.685	**0.380**
ABSI	0.569 (0.546–0.591)	0.08	0.550	0.552	0.102
**Males n = 1520**					
CUN-BAE	0.761 (0.737–0.785)	31.22	0.674	0.701	0.375
BMI	**0.773 (0.749–0.796)**	28.96	0.702	0.714	**0.416**
WHtR	0.761 (0.738–0.785)	0.61	0.675	0.7175	0.392
BRI	0.761 (0.738–0.785)	5.69	0.675	0.7175	0.392
ABSI	0.567 (0.538–0.596)	0.08	0.592	0.501	0.093
**Females n = 958**					
CUN-BAE	0.714 (0.682–0.747)	41.95	0.748	0.588	0.336
BMI	0.718 (0.686–0.750)	28.02	0.740	0.606	0.346
WHtR	**0.735 (0.704–0.767)**	0.61	0.702	0.666	**0.368**
BRI	**0.735 (0.704–0.767)**	5.77	0.702	0.666	**0.368**
ABSI	0.569 (0.554–0.626)	0.08	0.622	0.538	0.160

Adiposity index with the highest and area down the curve value in **bold**.

Abbreviations: ROC, receiver operating characteristic. CUN-BAE, Clínica Universidad de Navarra—Body Adiposity Estimator. BMI, body mass index. WHtR, waist-to-height ratio. BRI, body roundness index. ABSI, a body shape index.

The degree of agreement of the five adiposity indices analyzed for the purpose of identifying subjects with MetS are shown in S1 Table (additional file 1). In the stratified analysis, all values of the kappa index, except those corresponding to ABSI, are higher than 0.65, with the perfect correlation between BRI and WHtR standing out. In the global analysis, the degree of agreement between BMI, BRI and WHtR is also higher than 0.65; with CUN-BAE it is 0.40 and the maximum value reached with ABSI is 0.27.

### Association analysis

In the logistic regression analysis, all the adiposity indices, except for ABSI, show a similar probability of presenting MetS associated with the presence of obesity. The OR in the global analysis ranges from 5.50 (4.27–7.09) with CUN-BAE to 4.57 (3.81–5.49) with BMI. With males it is between 5.98 (4.70–7.60) with BMI and 5.51 (4.32–7.02) with CUN-BAE and with females between 4.15 (3.06–5.63) with CUN-BAE, 4.15 (3.09–5.58) WHtR and BRI, and 4.14 (3.07–5.58) with BMI (Table 4).

**Table 4:** Adjusted odds ratios of metabolyc syndrome by adiposity index.

a. Model 1. Odds Ratio Adjusted for Age and Gender						
	Global n = 2478		Males* n = 1520		Females* n = 958	
	OR (95% CI)	p value	OR (95% CI)	p value	OR (95% CI)	p value
CUN-BAE	**5.90 (4.63–7.51)**	<0.001	5.29 (4.22–6.63)	<0.001	4.42 (3.34–5.85)	<0.001
BMI	4.70 (3.96–5.58)	<0.001	5.83 (4.66–7.29)	<0.001	4.38 (3.33–5.77)	<0.001
WHtR	5.30 (4.44–6.32)	<0.001	5.93 (4.71–7.47)	<0.001	**4.78 (3.64–6.30)**	<0.001
BRI	5.32 (4.46–6.34)	<0.001	**5.99 (4.75–7.54)**	<0.001	**4.78 (3.64–6.30)**	<0.001
ABSI	1.78 (1.50–2.10)	<0.001	1.66 (1.34–2. 05)	<0.001	1.96 (1.51–2.54)	<0.001
**b. Model 2**. Odds Ratio Adjusted for Age, Gender, Smoking Status, Alcohol consumption, Adherence to the Mediterranean diet and Physical Activity Status						
CUN-BAE	**5.79 (4.54–7.39)**	<0.001	5.28 (4.21–6.24)	<0.001	4.18 (3.14–5.56)	<0.001
BMI	4.60 (3.87–5.46)	<0.001	5.81 (4.64–7.28)	<0.001	4.13 (3.12–5.46)	<0.001
WHtR	5.21 (4.36–6.23)	<0.001	5.96 (4.72–7.53)	<0.001	**4.54 (3.43–6.01)**	<0.001
BRI	5.23 (4.37–6.25)	<0.001	**6.02 (4.76–7.60)**	<0.001	**4.54 (3.43–6.01)**	<0.001
ABSI	1.78 (1.50–2.10)	<0.001	1.66 (1.33–2. 05)	<0.001	1.96 (1.51–2.55)	<0.001
**c. Model 3**. Odds Ratio Adjusted for Age, Gender, Smoking Status, Alcohol consumption, Adherence to the Mediterranean diet, Physical Activity Status, Antihypertensive drugs, Antidiabetic drugs and Lipid-lowering drugs						
CUN-BAE	**5.50 (4.27–7.09)**	<0.001	5.51 (4.32–7.02)	<0.001	4.15 (3.06–5.63)	<0.001
BMI	4.57 (3.81–5.49)	<0.001	**5.98 (4.70–7.60)**	<0.001	4.14 (3.07–5.58)	<0.001
WHtR	5.02 (4.16–6.06)	<0.001	5.93 (4.63–7.58)	<0.001	**4.15 (3.09–5.58)**	<0.001
BRI	5.04 (4.18–6.09)	<0.001	5.96 (4.66–7.63)	<0.001	**4.15 (3.09–5.58)**	<0.001
ABSI	1.69 (1.41–2.02)	<0.001	1.65 (1.31–2. 07)	<0.001	1.81 (1.37–2.39)	<0.001

Adiposity index with the highest significant OR value in **bold**.

* In the analysis in males and in females the gender was not used as adjustment variable

Abbreviations: OR, odds ratio. CI, confidence interval. CUN-BAE, Clínica Universidad de Navarra—Body Adiposity Estimator. BMI, body mass index. WHtR, waist-to-height ratio. BRI, body roundness index. ABSI, a body shape index.

Cut points used with the different adiposity index: Global: CUN-BAE: 33.15, BMI: 28.73, WHtR: 0.613, BRI: 5.67 and ABSI: 0.0834; Males: 31.22, BMI: 28.96, WHtR: 0.611, BRI: 5.69 and ABSI: 0.0835 and females: 41.95, BMI: 28.02, WHtR: 0.615, BRI: 5.77 and ABSI: 0.0816.

## Discussion

The present study, conducted with Spanish subjects at intermediate cardiovascular risk, suggests that both the association of the adiposity indices with MetS and their ability to identify MetS subjects is similar with all indices analyzed, both in the global analysis and by gender, apart from ABSI. The probability of developing MetS associated with the presence of obesity ranged in the global analysis with the best-fit model from 5.50 with CUN-BAE to 4.57 with BMI, being lower in females than in males. These results are confirmed in the ROC curve analysis.

The results of this study are new and may be of important clinical relevance since it is the first study that compares the capacity of five adiposity indices to identify subjects with MetS in a population with intermediate cardiovascular risk. The results suggest that, except for ABSI, they may all be useful in clinical practice for identifying subjects at higher risk of developing MetS.

Over recent years, several studies have assessed the capacity of different adiposity indices to identify subjects with MetS, without obtaining conclusive results. The results coincide with those published in other studies conducted with American adolescents or in the general oriental population in which BMI, WC and WHtR behaved similarly in identifying subjects with MetS [4, 5, 35]. However, other studies conducted with oriental adults [30, 36], or overweight/obese children and adolescents [30, 36] have shown that WHtR had greater power to identify subjects with MetS, but these did not include CUN-BAE nor ABSI among the indices analyzed. On the other hand, the Women’s Health Initiative Study [37] involving 2672 postmenopausal females showed that BMI had greater predictive power than WHtR when identifying MetS.

The AUROC results obtained by the adiposity indices for identifying subjects with MetS coincide with those published in other studies [4, 5, 7]. Similarly, several studies [5, 38, 39], although not all [39], have reported that ABSI was a weak indicator of subjects with MetS.

Furthermore, previous studies conducted in Asian and American populations [38,39] showed that the relationship of the different components of MetS with the adiposity indices analyzed was stronger in females than in males [19, 40, 41], data that coincide in part with the results of the present study. The reason for this sex difference is unclear, although differences in anatomy, physiology, metabolism, and sex hormones may offer a partial explanation [36]. There are similar gender differences with the optimal cut-off points for the five adiposity indices analyzed, which suggests that gender-specific reference values should be used to identify subjects with a higher risk of MetS.

Regarding the adiposity indices less frequently studied in identifying subjects with MetS, we must note that the CUN-BAE estimates the percentage of body fat and it is the excess body fat which is chiefly responsible for the complications associated with obesity [42, 43]. Although in our study CUN-BAE is not superior to other measures in predicting MetS, there are previous studies that suggest that the determination of body fat percentage can better predict MetS [12, 44]. The BRI was developed to measure body fat and the percentage of visceral adipose tissue by using WC in relation to height, which allows the estimation of the shape of the human body as an ellipse or oval [18]. The BRI correlates with MetS components and identifies MetS as other adiposity indices do. The equal predictive power (identical areas under the ROC curve) of BRI and WHtR which this study found among females has been described in previous studies [19, 20, 45]. The Spearman coefficient revealed an extremely close relationship between the two indices (r = 1).

Finally, ABSI was created as a quantitative measure to estimate the health of body shape regardless of body size, so ABSI predicts premature mortality better than BMI or WC [15]. Our results suggest that, compared to the other four indices used in this study, ABSI should not be used to predict MetS. A possible explanation for the non-concordant findings between our data and those of Krakauer et al. [15], are the different end point variables (mortality and MetS), the characteristics of the subjects analyzed (general population and subjects of intermediate cardiovascular risk) and height, which was 1.70 meters in the study by Krakauer et al. [15], while in ours it was 1.65 meters, indicating that height could influence ABSI’s ability to identify MetS. Future studies should therefore further investigate the limits of ABSI and the impact of body height on the calculation of ABSI.

The main limitation of our study is its transversal design, which does not allow us to establish causal relationships or their direction. Secondly, the population in this study was ethnically homogeneous, since all the patients in this study were Caucasians at intermediate cardiovascular risk, thus potentially limiting the generalizability of our findings. However, its strong points include the large sample analyzed and the fact that as adjustment variables we have used those which according to other authors [19] can influence the different adiposity indices, such as age, gender, sociodemographic variables, dietary intake, physical activity and consumption of alcohol and tobacco, which could affect the strength of the associations.

## Conclusions

All adiposity indices, except for ABSI, show an association with MetS and similar ability to detect subjects with MetS among people with intermediate cardiovascular risk.

## Supporting information

S1 Table Degree of agreement between the adiposity indices to identify subjects with MetS. Kappa index.
(DOC)

S1 File Data file. (SAV)

## Abbreviations

ABSI: adiposity with body shape index
ABSI: body shape index
AUROC: ROC curve
BMI: body mass index
BRI: body roundness index
CUN-BAE: Clínica Universidad de Navarra—body adiposity estimator
DQI: diet quality index
MARK: MediAte Risk management
METs: mean metabolic
ROC: Receiver-operating characteristic
SBP: systolic blood pressure equivalents
WC: Waist circumference
WHtR: waist-to-height ratio
