# Supplementary material for: Capacity adiposity indices to identify metabolic syndrome in subjects with intermediate cardiovascular risk (MARK study)
Source: PLoS One. 2019 Jan 25;14(1):e0209992. doi: 10.1371/journal.pone.0209992 (PMC6347134; doi:10.1371/journal.pone.0209992)
Supplement: S1 Table — Kappa index. (DOC) [file pone.0209992.s001.doc]

**Table 1S. Degree of agreement between the adiposity indices to identify subjects with MetS. Kappa index.**

| **Global** | **BMI** | **WHtR** | **BRI** | **ABSI** |
| --- | --- | --- | --- | --- |
| **CUN-BAE** | 0.396* | 0.406* | 0.401* | 0.109* |
| **BMI** | 1 | 0.653* | 0.654* | 0.034** |
| **WHtR** |  | 1 | 0.990* | 0.270* |
| **BRI** |  |  | 1 | 0.168* |
| **Males** |  |  |  |  |
| **CUN-BAE** | 0.880* | 0.684* | 0.679* | 0.018 |
| **BMI** | 1 | 0.651* | 0.646* | 0.018 |
| **WHtR** |  | 1 | 0.995* | 0.280* |
| **BRI** |  |  | 1 | 0.280* |
| **ABSI** |  |  |  | 1 |
| **Females** |  |  |  |  |
| **CUN-BAE** | 0.958* | 0.685* | 0.685* | 0.020 |
| **BMI** | 1 | 0.671* | 0.671* | 0.022 |
| **WHtR** |  | 1 | 1 | 0.272* |
| **BRI** |  |  | 1 | 0.272* |

Abbreviations: CUN-BAE, Clínica Universidad de Navarra - Body Adiposity Estimator. BMI, body mass index. WHtR, waist-to-height ratio. BRI, body roundness index. ABSI, a body shape index.

Cut points used with the different adiposity index: Global: CUN-BAE: 33.15, BMI: 28.73, WHtR: 0.613, BRI: 5.67 and ABSI: 0.0834; Males: CUN-BAE: 31.22, BMI: 28.96, WHtR: 0.611, BRI: 5.69 and ABSI: 0.0835 and females: CUN-BAE: 41.95, BMI: 28.02, WHtR: 0.615, BRI: 5.77 and ABSI: 0.0816.

*P<0.01. **P<0.05
